# Determining the Associations between Dietetic-Related Activities and Undergraduate Dietetic Students’ General Cultural Knowledge, Attitudes, and Beliefs

**DOI:** 10.3390/nu11061202

**Published:** 2019-05-28

**Authors:** Jeanette Mary Andrade

**Affiliations:** Department of Food Science and Human Nutrition, University of Florida, Gainesville, FL 32611, USA; jandrade1@ufl.edu; Tel.: +1-352-294-3975; Fax: +1-352-392-9467

**Keywords:** college students, cultural competency, instrument validation, cultural activities

## Abstract

Background: As required by the Accreditation Council for Education in Nutrition and Dietetics, undergraduate dietetic programs need to include classroom learning activities to support cultural competence among dietetic students. Though these activities vary in terms of type, length, and engagement, it is not known the impact these activities have on students’ general knowledge, attitudes, and beliefs (KAB) towards cultural aspects. Therefore, the study’s purpose was two-fold: (1) validate a general cultural KAB instrument for dietetic students, and (2) determine associations among dietetic-related cultural activities and students’ KAB. Methods: A general KAB instrument was developed based on a literature review and dietetic curriculum. The original instrument (34 items) consisted of two dimensions (i.e., knowledge and attitudes/beliefs) that was reviewed by content matter experts (*n* = 4) and a focus group with dietetic graduate students (*n* = 6), resulting in a 41-item tool. This instrument was further piloted in a diverse population of undergraduate dietetic students across the United States. Exploratory Factor Analysis (EFA) and Cronbach alpha (*α*) for internal consistency were conducted. Multiple linear regressions and Spearman correlation analyses determined associations between demographics, activities, and KAB scores. Statistical significance was determined at *p* < 0.05. Results: Students (*n* = 187) completed the questionnaire. From the EFA, the Knowledge dimension included 12 items and the Attitudes/Beliefs dimension included 10 items. Internal consistency for the overall instrument (*α* = 0.86), Knowledge (*α* = 0.93), and Attitudes/Beliefs (*α* = 0.74) was high. Students’ cultural knowledge was associated (*r* = 0.30; *p* < 0.05) with cultural-related activities. Similarly, students who had lived or studied abroad had better attitudes and beliefs towards cultural aspects. Conclusions: The KAB had good validity. Cultural learning activities enhanced cultural knowledge, however to a lesser extent influenced the attitudes and beliefs of dietetic students.

## 1. Introduction

The United States (US) continues to expand in its number of ethnic and racial groups. As projected, by 2044 more than 50% of the US will belong to a minority group and by 2060, more than 20% of the US population will be foreign born [1]. This trend is reflected in Healthy People 2020 as one goal is to “achieve health equity, eliminate disparities, and improve the health of all groups” [2]. Unfortunately, health disparities among all age groups still exist for certain ethnic/racial groups, sexes, geographical locations (rural), and socioeconomic statuses and health professionals. As a result, the Accreditation Council for Education in Dietetics and Nutrition (ACEND) [3] has responded by requiring all accredited didactic programs to incorporate cultural components (e.g., language, thoughts, communications, actions, customs, beliefs, values, and institutions of racial, ethnic, religious, or social groups) into their curriculum to enhance dietetic students’ cultural competence [3]. ACEND aims at preparing future dietitians to provide a higher standard of care to a more diverse population and subsequently reduce health disparities [3].

Prior to the discussion of cultural competence, it is necessary to define culture. Depending on the research context, several definitions of culture exist. For this study, Leininger’s (1991) definition of culture will be used. She defines it as “the learned, shared, and transmitted values, beliefs, norms and lifeways of a particular culture that guides thinking, decisions, and actions in patterned ways and often intergenerationally” (p. 47) [4]. Just as culture is difficult to define, cultural-competence is a complex concept that, in the health professional field, is commonly defined as the aptitude of a practitioner to respond to a patient’s cultural knowledge, attitudes, and beliefs towards healthcare [5,6]. This idea of cultural-competence goes beyond race and ethnicity and encompasses socioeconomic and educational statuses, occupation, political beliefs, residence (urban versus rural), sexual orientation, among others [7]. Many cultural-competence models exist, however one that is commonly used for patient care is Campinha-Bacote’s [8]. In her model, she indicates that cultural competence is an ongoing process that consists of five constructs: awareness, skill, sensitivity, knowledge, encounter, and desire. Cultural awareness is knowingly acknowledging one’s own cultural background to prevent biases towards other cultures. Cultural skill refers to both verbal and nonverbal communication that other cultures can comprehend [8,9]. Cultural knowledge is the educational base of healthcare practitioners’ understanding of the various cultural and ethnic traits associated with patient’s viewpoints toward health and illness. A cultural encounter is an interaction between a practitioner and patient who has a different cultural background to the practitioner. During this interaction, the pair communicate in different forms such as face-to-face, the internet, or telephone during a short or long period of time. The frequent interactions between the two are correlated with the practitioner understanding the patient’s cultural background [9,10]. Cultural desire is the motivation to be educated, skilled, and aware of various cultures [8,9]. The desire to expose oneself to patients from different cultural backgrounds begins the process of becoming culturally competent [11]. In essence, a practitioner must not only be cognitively aware of the patient’s culture, however they must also be able to respond appropriately [12]. Even though practitioners may self-identify themselves as being culturally-competent, results from studies indicate that there is little to no improvement in patient health outcomes [13,14,15]. In this case, practitioners perhaps have knowledge and understanding of cultures, however not necessarily positive attitudes and beliefs towards them.

ACEND requires the incorporation of experiential learning opportunities in the classroom to support cultural competence among dietetic students [3]; for example, developing a type 2 diabetes program for low-income Hispanic adolescents or creating meal plans for homebound elders who avoid pork and caffeine due to religious reasons. Furthermore, the dietetic faculty can incorporate culture in a variety of methods in the course such as case studies, projects, or study abroad opportunities to support students’ cultural competence. Results from previous studies indicate that incorporating a social and active/experiential learning activity within dietetic courses enhances students’ knowledge and awareness towards cultural competence [16,17]. These studies, however, employed different survey instruments that were specific to their objectives. Thus, it is not known the extent to which these course activities, regardless of whether there is a social and active/experiential learning component or not, influenced students’ general cultural knowledge, attitudes, and beliefs (KAB). Therefore, the purpose of this study was two-fold: (1) validate a general cultural KAB instrument for undergraduate dietetic students using an established conceptual framework of culture, and (2) determine the associations between the exposure to class activities with culturally-diverse themes and the general cultural KAB score among a diverse population of undergraduate dietetic students. The main hypothesis for this study was that exposure to various class activities with culturally diverse themes would positively influence the KAB of dietetic undergraduate students.

## 2. Materials and Methods

### 2.1. Development of the General Cultural Knowledge, Attitudes, and Beliefs Instrument

For the first version of this instrument, the items were developed based on a literature review of pre-existing general KAB instruments for the curriculum [16,18], the definition of culture [4], descriptors of culture [19,20], dietetic clinical practices, and activities commonly found within undergraduate dietetic courses. Choices for the course activities were based on the author’s review of the United States’ undergraduate dietetic syllabi (*n* = 81) which were publicly available online and had listed cultural activities to be completed in the course and were considered active courses (taught either in Fall, Spring, and Summer of 2016 or 2017 or Spring 2018). The author reviewed the syllabi within the areas of community nutrition (*n* = 9), diet planning (*n* = 2), dietetic related food/clinical cultural courses (*n* = 5), food/clinical management (*n* = 15), fundamentals of nutrition (*n* = 5), medical nutrition therapy (*n* = 15), nutrition counseling (*n* = 10), nutrition education (*n* = 7), and nutrition in the life cycle/life span (*n* = 13). Initially, a total of 11 activities were listed through the syllabi review (e.g., discussions, presentations, and service learning). The original instrument consisted of four dimensions with a total of 34 items—knowledge (*n* = 6), attitudes and beliefs (*n* = 12), dietetic program addressing culture (*n* = 3), perceptions of dietetic professors’ cultural knowledge (*n* = 7), culture integrated into specific courses and types of activities completed (*n* = 2), and demographics (*n* = 4). Initially, a 4-point Likert scale ranging from strongly disagree (1) to strongly agree (4) was used for the knowledge, attitudes, and beliefs portion. This scale was consistent with other cultural KAB instruments [21,22,23,24] in which the neutral response was eliminated for participants to cognitively explore their opinions towards these statements [25,26,27,28]. Finally, the statements for the types of cultural activities were completed and demographic information were structured on a multiple-choice format. 

### 2.2. Stage One

#### Design and Participants

Content matter experts (CME) (*n* = 4) reviewed the instrument for content validity. The content matter experts had between 5–15 years of educating within the undergraduate dietetic field, had validated survey instruments in the past, and were aware of the ACEND standards to assess cultural competence. On a scale from 1 (not at all) to 4 (completely), CMEs rated each statement for clarity, relevance, and ambiguity. Additionally, CMEs provided strengths, weaknesses, and suggestions to enhance each statement. Considering that the content domain that was rated was narrowly defined and the rater agreement was high, no additional raters were identified to validate the content of the instrument [29]. Results from the CMEs provided suggestions to enhance the context of the knowledge, attitudes, and beliefs statements and undergraduate dietetic course activities. Modifications were made to clarify that the students should focus on dietetic professors as opposed to professors in general, adding four additional questions to the knowledge section, clarifying the completion of dietetic courses that included culture, and including two additional dietetic-specific activities. 

A focus group which comprised of graduate dietetic students (*n* = 6) took place to validate the modified instrument for face and content validity. The instrument was further modified and expanded to include a total of 41 items (see Supplementary Table S1)—knowledge (*n* = 7), attitudes and beliefs (*n* = 12), dietetic program addressing culture (*n* = 2), non-dietetic courses addressing culture (*n* = 1), perceptions of dietetic professors’ cultural knowledge (*n* = 4), and culture integrated into specific courses, specific course objectives focusing on culture and types of activities completed (*n* = 3), and demographics (*n* = 12). A total of 11 activities remained on the course activities, although examples were included to clarify the listed activities. For example, “interactive case studies (e.g., talking with a patient, role playing).” The demographic statements were expanded to include languages that participants were able to speak, read, and write fluently, if participants lived or studied abroad, if they were double-majors and/or minors, and the content area of these majors/minors. The definition of culture and components of culture were also included in the instrument to minimize misinterpretation. The Likert scale was expanded to six responses which included mildly disagree (3) and mildly agree (4) as focus group participants did not completely disagree or agree with statements and preferred the choice responses as opposed to a neutral response. Table 1 summarizes the dimensions of the instrument and the number of items.

### 2.3. Stage Two

#### Design and Participants

The final version of the general KAB instrument was developed in Qualtrics (Seattle, WA, USA), an online survey platform, and an email was sent to the Didactic Programs in Dietetics (DPD) directors (*n* = 213) across the United States. The email informed the directors about the purpose of the study and provided them with an email to send to their undergraduate dietetic students. The student email informed them about the purpose of the study and included the link to complete the instrument along with an informed consent. Based on 2018 enrollment data from ACEND, approximately 12,972 students may have been reached to complete this instrument [30]. This, though, depended on if directors provided it to all their students or selected those to participate. Survey data were collected online anonymously through Qualtrics. No compensation was provided for participation. Two reminder emails were sent to DPD directors to encourage participation at one and two months post initial email. Both study stages one and two of the study were approved by the Institutional Review Board at the University of Florida. 

### 2.4. Statistical Analysis

Frequency counts and percentages were tabulated for knowledge and attitudes and beliefs scores, course assignments and activities, and demographic variables. Exploratory factor and parallel analyses were performed on 26 items of the general KAB instrument—knowledge (*n* = 7), attitudes and beliefs (*n* = 12), dietetic program addressing culture (*n* = 2), non-dietetic courses addressing culture (*n* = 1), and perceptions of dietetic professors’ cultural knowledge (*n* = 4)—as there were a minimum of 100 participants [31,32,33]. The principal axis factoring [34] was completed to extract the factors, which was followed by a varimax rotation and scree plot. Based on the scree plot, factors were retained above the “break” point [35]. Only dimensions and items that did not cross-load and had factor loadings of 0.32 and above were retained [36].

Internal consistency reliabilities of the knowledge, attitude, and beliefs statements were determined through Cronbach alpha coefficients. Considering that one dimension focused on attitudes and beliefs, Cronbach alphas of 0.60 and above for the instrument and the individual items were considered adequate [37,38]. One knowledge and two attitude and beliefs statements were reverse coded. Sub-scores were obtained from the knowledge and attitude and beliefs dimensions and were averaged separately to report a total score from each dimension. 

Two multiple linear regression analyses were conducted to examine the associations between demographics, cultural activities, and scores from the knowledge and attitudes and beliefs dimensions. Multiple linear regressions examined the confounding factors (demographics) and isolated the relationship of interest (knowledge or attitudes and beliefs) [39]. The average knowledge score was regressed onto the number of cultural activities and demographics (Model 1). Additionally, the average attitudes and beliefs score was regressed onto the number of cultural activities and demographics (Model 2). The effect size classification suggested by Cohen (1988) [40] was used to present the strength of *R*^2^. The strength of *R*^2^ was classified as small, medium, and large when *R*^2^ = 0.01, 0.09, and 0.25, respectively [34,41].

*Y_1_* = *b*_0_ + *b*_1_X_1_ + *b*_2_X_2_ +… + *b*_k_X_k_(Model 1)

Where,

*Y_1_* = Knowledge score*b*_0_, *b*_1_, and *b*_k_ =Estimate regression parametersX_1_, X_2_, and X_k_ =k predictors (number of cultural activities and demographics)

*Y_2_* = *b*_0_ + *b*_1_X_1_ + *b*_2_X_2_ +… + *b*_k_X_k_(Model 2)

Where,

*Y_2_* = Attitudes and Beliefs scores *b*_0_, *b*_1_, and *b*_k_ =Estimate regression parameters X_1_, X_2_, and X_k_ =k predictors (number of cultural activities and demographics)

A Spearman correlation (Pearson chi-square) was used to measure the strength of the association between the general cultural KAB scores and the individual cultural activities. Statistical significance was determined at *p* < 0.05. All statistical analyses were conducted using the Statistical Package for Social Sciences (version 25, SPSS, Inc, Chicago, IL, USA). 

## 3. Results

### 3.1. Study Population Characteristics

A total of 302 students initially consented to the study. However, only 187 students’ feedback were included in this study due to missing data from the others. As consistent with the demographics of the US dietetic population [42], the majority were female (91.9%) and white (64.5%). A large portion were seniors (48%), spoke only English and no other language (72%), were from the East North Central region of the United States (26.2%), and had neither studied abroad nor lived abroad (69%) (see Table 2).

Regarding dietetic course activities related to culture, 31% of students indicated that they participated in 2–3 cultural course activities. For specific individual course activities related to culture, the majority (63.6%) participated in discussions (see Table 3).

### 3.2. Factor Analysis of the General Cultural KAB

Based on the 26 items that underwent exploratory factor analysis and further primary axis factoring, a total of four factors were retained. From the minimum threshold level of 0.32 and no cross-loadings, the following factor statement items were removed: two from attitudes and beliefs; “I am less patient with people from different cultural backgrounds than my own” and “I feel comfortable working with people from different cultural backgrounds than my own”. Additionally, two were removed from perceptions of dietetic professors’ cultural knowledge: “My dietetic professors are comfortable discussing cultural issues in the classroom” and “My dietetic professors respect students from different cultures”. The first factor contained 12 items, which included knowledge and educational experiences, subsequently identified as Knowledge. The second factor contained 10 items and combined attitudes and beliefs towards coursework and interactions with individuals from different cultural backgrounds and was thus identified as Attitudes and Beliefs. Although not all items loaded together, as initially developed, the factors that emerged were consistent with the conceptual development of this instrument (see Table 4). 

The average item means and standard deviations for the total scale are displayed in Table 5. For the knowledge statements, students, on average, agreed (4.5 ± 0.89). For the attitudes and beliefs statements, students, on average, agreed (4.45 ± 0.41). However, for three statements—“before speaking with someone, I have pre-conceived notions about their culture”; “my cultural background influences how I behave in the classroom”; and “my own cultural beliefs may influence the decisions I make in patient simulations”, students, on average, mildly disagreed with those statements: 3.74 ± 1.15, 2.99 ± 1.32, and 3.49 ± 1.38, respectively.

Cronbach’s alpha coefficients were computed to assess internal consistency reliability for the instrument. For the overall instrument, reliability was at *α* = 0.86, which indicates good reliability. Based on the new dimensions, the knowledge dimension (*n* = 12) had a reliability of *α* = 0.93 and the reliability of the attitudes and belief dimension (*n* = 10) was *α* = 0.74. Thus, based on the reliability of the dimensions, the instrument was deemed acceptable (see Table 5). 

### 3.3. Associations between General KAB Scores and Cultural Activities

The multiple linear regression of knowledge scores on the variables shown in Table 6 for Model 1 accounted for a small proportion (5.1%) of the variance explaining the implementation degree (Adjusted *R*^2^ = 0.051, F-test (7, 178) = 2.4, Mean Squared Error (MSE) = 0.87, *p* = 0.02). Results indicated that the number of cultural activities (more than two) (*β* = 0.05, *p* = 0.001) was positively associated with knowledge scores. In other words, students exposed to more cultural activities positively agreed with the knowledge statements. No associations were seen between demographics and students’ cultural knowledge. The multiple linear regression based on the attitudes and beliefs scores on the variables shown in Table 6 for Model 2 accounted for a small proportion (4.7%) of the variance explaining the implementation degree (Adjusted *R*^2^ = 0.047, F-test (7, 178) = 2.3, MSE = 0.40, *p* = 0.03). Results showed that students who studied or lived abroad (*β* = −0.07, *p* = 0.01) were positively associated with the attitudes and beliefs scores. This indicated that students who lived or studied abroad had positively agreed with the attitudes and beliefs statements. There were no other associations seen among the demographics and number of cultural activities and students’ cultural attitudes and beliefs scores.

**Figure 1:**
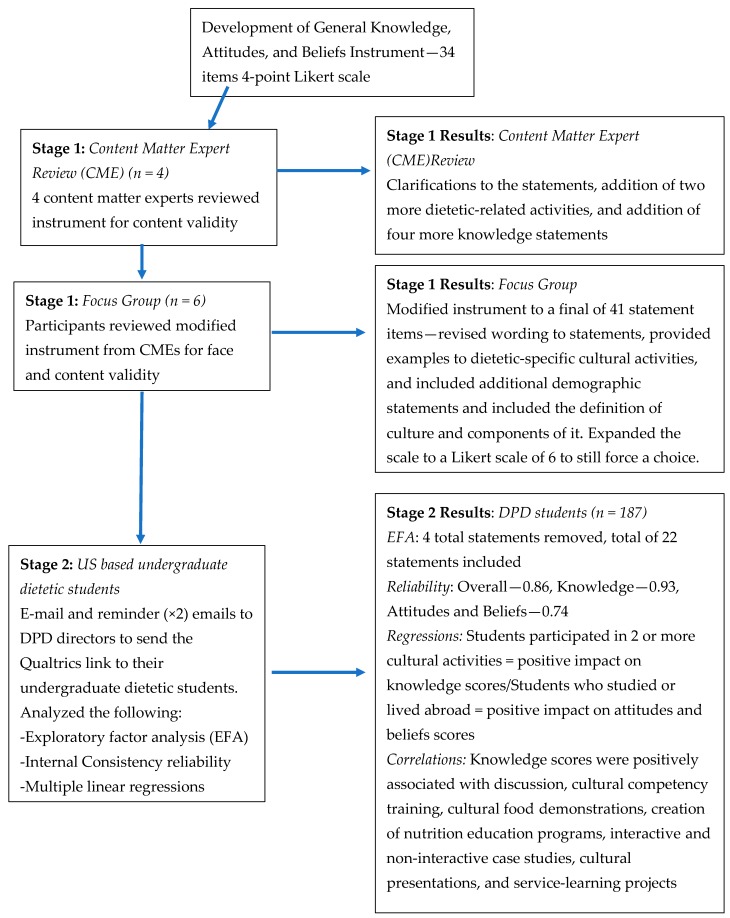
Stages of the study and key findings.

Based on the Spearman correlation, several strong positive associations were seen between the dietetic students’ general knowledge scores and the following individual activities (*p* < 0.05): discussion, cultural competency training, cultural food demonstrations, creation of nutrition education programs, interactive and non-interactive case studies, cultural presentations, and service-learning projects. There were no associations seen between knowledge scores and the following cultural activities: internship (*r = 0*.134, *p* = 0.07) or study abroad experience (*r* = 0.09, *p* = 0.23). There were no associations found among general attitudes and beliefs scores and individual cultural activities (see Table 7). An overall description of the stages of the study and key findings are found in Figure 1. 

## 4. Discussion

The purpose of this study was to validate a general cultural KAB instrument for undergraduate dietetic students and to determine associations among dietetic-related activities and undergraduate dietetic students’ KAB. Data from the exploratory factor analysis (EFA) and Cronbach alphas revealed that the instrument had fair construct validity and good internal consistency reliability, even if through the EFA, items loaded on different dimensions than initially devised. Even though this instrument had fair validity and good reliability, the instrument should undergo additional analysis to ensure the consistency of the instrument.

Results from the multiple linear regression and correlation analyses showed that undergraduate dietetic students’ knowledge scores were positively associated with specific cultural activities, mainly those considered interactive activities such as class discussions, cultural food demonstrations, and interactive case studies. Other studies support this notion that students who actively participate in their learning enhance their knowledge and understanding of cultural-competence [16,17]. In active learning, the educator functions as more of a facilitator as opposed to a teacher or lecturer [43,44,45]. In this approach, students improve their critical thinking skills and ability to perform an activity at higher cognitive levels [44,45,46,47]. However, in this study, no associations were seen among students’ cultural knowledge scores and internship and study abroad. These results could have been because a small number of participants, 3% and 6%, did an undergraduate internship and participated in a dietetic specific study abroad. This was also evidenced by McArthur and colleagues’ study (2011) [16] in which few undergraduate dietetic students participated in internships and study abroad. Even though didactic accredited programs need to comply with ACEND’s standards, the types of activities to meet those standards are up to each individual program [3,6,48]. Therefore, programs may not require students to complete an undergraduate internship or a dietetic-specific study abroad. Based on other health professional studies, these activities may be a good means to further enhance not only the knowledge, however also the ability of a dietetic student to be more culturally competent through their behaviors [49,50,51]. Also, these activities may be integrated within the current didactic program without overwhelming the student, such as creating an international online class in which students from a domestic institution and students from a foreign institute participate in virtual web-based discussions/lectures [52,53]. Even though this method limits the rich experience seen in internships and study abroad programs, it allows students to understand more about a culture.

Students’ attitudes and beliefs were not associated with individual cultural activities. This is in opposition to other studies that showed if students were exposed to different interactive cultural activities, their attitudes towards different cultures improved [16,17,48,50]. McArthur and colleagues (2011) [16] determined cultural knowledge, attitudes, and experiences of nutrition and dietetic students (*n* = 283) through a Cultural Nutrition Knowledge Test (CNKT) designed by the researchers. Students were presented with various scenarios and had to select the answer that best fit. The knowledge portion of this instrument was a multiple-choice test based on these scenarios. Results showed that students who were exposed to multiple cultural activities throughout their dietetic curriculum had higher knowledge and attitude scores compared to students who were exposed to limited cultural activities. Hack and colleagues (2015) developed a six-part questionnaire that contained a total of 24 questions to examine the cultural competence of dietetic students (*n* = 133) to identify methods to enhance their current curriculum. Results showed that the more cross-cultural activities students were exposed to, the higher the cultural competence scores compared to those students who had indicated less activities. Additionally, their findings showed that if dietetic students completed a cultural foods course, they indicated higher scores of cultural knowledge, skills, attitudes, and awareness as opposed to those students who did not complete that course. Finally, Wright and Lundy (2015) and Werremeyer and colleagues (2012) conducted mixed-method studies to determine the impact a service-learning project had on students’ cultural knowledge, attitudes, and awareness. Both of these studies used cultural instruments that were previously validated, such as the Health Professions Schools in Service to the Nation (HPSISN) instrument [17] and the Inventory for Assessing the Process of Cultural Competence—Student Version (IAPCC-SV) instrument [50]. Both studies demonstrated that students enhanced their knowledge, attitudes, and awareness of cultural groups. However, both of these studies had a small sample size, 6 and 4, respectively, thus caution needs to be exercised when interpreting these results. Regardless, future studies should focus on the impact that specific interactive cultural activities have on students’ cultural knowledge, attitudes, and beliefs and the lasting impact.

Although, findings from this study showed that, on average, students mildly disagreed on three statements, which were (1) their attitudes and beliefs towards if they have preconceived notions about someone from a different culture, (2) how they behaved in the classroom when discussing other cultures, and (3) their perceptions about handling a patient from a different culture. Even though there were no associations between attitudes and beliefs and cultural activities, at least in this study, these results demonstrate that the cultural activities were having some positive impacts on their cultural attitudes and beliefs. Several studies have indicated similar results in which health professionals who were exposed to cultural competency activities, such as training, held positive attitudes and beliefs towards different cultural groups [54,55,56]. Jongen and colleagues (2018) [55] conducted a systematic review to determine the impact cultural competency interventions had on practitioners’ knowledge, attitudes, skills, and behavior. Five of the 16 studies, which focused on attitudes and beliefs, showed that cultural competency training that was categorical (starting from knowledge: fundamentals of cultural competency to higher order thinking: practicing with cultural competency) or cross-cultural approaches (working with individuals from certain racial/ethnic, etc. backgrounds) resulted in positive attitudes and beliefs towards culture [55]. Therefore, undergraduate dietetic programs should continue to incorporate various and numerous cultural interactive activities in dietetic courses such as cultural presentations, training, and discussions to positively impact not only knowledge, however also attitudes and beliefs. 

This current study demonstrated that students who lived abroad or studied abroad had a positive association with cultural attitudes and behaviors scores. Study abroad programs are a mechanism to enhance cultural competence as they provide an immersive experience for students to interact with diverse populations [49,57,58]. Even though few students participated in a dietetic-specific study abroad program, respondents had participated in a general study abroad (11%), lived abroad (8.7%), or did both (5.5%) during their four-year college experience. Dietetic students who live or study abroad may be at a greater advantage when working with patients from different backgrounds compared to students who do not participate in study abroad. When students study abroad, they are in an unfamiliar environment, thus it allows students to explore their own values, beliefs, and biases. Due to this, when the students return home, they embrace diversity and are more confident when interacting with individuals from a different cultural background than themselves [59,60]. To encourage more dietetic students to participate in a study abroad, in the dietetic curriculum, the dietetic faculty could connect with other institutions abroad within their classes.

Overall, participants in this study had high mean scores through the knowledge and attitudes and beliefs dimensions of the general KAB instrument. Associations were found between knowledge scores and specific dietetic-related cultural activities, although none were found between the attitudes and beliefs scores. However, a relationship was identified between attitudes and beliefs and students either living or studying abroad. Considering that no data exists on students’ general KAB prior to starting their dietetic program, it is possible that dietetic-specific cultural activities did increase with program exposure. Further studies involving pre- and post-program measures using the general KAB are warranted. 

Overall, there is a need to develop cultural-competent dietitians within the United States. As the goal of the dietetic program is to educate diverse dietetic students on culturally competent practices, it is critical to monitor and evaluate students’ progress towards this goal. The general cultural KAB is a tangible method to determine if the dietetic cultural activities within the classroom have an impact on students’ cultural competency. Based on the findings from this study, the general cultural KAB provided valid and reliable results in the study population, however further research on the use of the instrument in diverse populations would be beneficial.

## Figures and Tables

**Table 1:** Initial Categories for General Knowledge Attitudes and Beliefs Instrument.

Category	No. of Items
Attitudes and Beliefs	12
Knowledge	7
Perceptions of dietetic professors’ cultural knowledge	4
Dietetic program addressing culture	2
Non-dietetic courses addressing culture	1

**Table 2:** Demographics (*n* = 187).

Demographics	No. of Responses (%)
**Gender**	
Male	12 (6.4%)
Female	171 (91.4%)
Other	2 (1.1%)
Prefer not to respond	2 (1.1%)
**Ethnicity**	
Asian	15 (8%)
African-American	8 (4.3%)
Hispanic/Latino	26 (13.9%)
White	120 (64.2%)
2 or more	10 (5.3%)
Other	2 (1.1%)
Prefer not to respond	6 (3.2%)
**Age**	
18–19 years	20 (10.7%)
20–21 years	63 (33.7%)
22–24 years	54 (28.9%)
25 years and above	47 (25.1%)
Prefer not to respond	3 (1.6%)
**Year in School**	
Freshman	9 (4.9%)
Sophomore	17 (9.1%)
Junior	54 (28.8%)
Senior	90 (48.1%)
Post-baccalaureate	17 (9.1%)
**Studied/Lived Abroad**	
Studied abroad	24 (12.8%)
Lived abroad	19 (10.2%)
Both	12 (6.4%)
Neither	129 (69%)
Prefer not to respond	3 (1.6%)
**Languages—Speak Fluently**	
English only	134 (72%)
English plus another language	47 (25%)
English plus two or more other languages	6 (3%)
**Languages—Write/Read Fluently**	
English only	134 (72%)
English plus another language	47 (25%)
English plus two or more other languages	6 (3%)
**Double Major**	
Yes	11 (5.9%)
No	176 (94.1%)
**Other Major Concentration**	
Biology/Microbiology	2 (18.2%)
Economics	1 (9.1%)
Exercise science	4 (36.4%)
Food science	1 (9.1%)
No response	3 (27.2%)
**Minor**	
Yes	53 (28.3%)
No	134 (71.7%)
**Minor Concentration**	
Anthropology/Psychology/Sociology	11 (21.2%)
Business Administration/International Administration/Tourism	5 (9.6%)
Biology/Chemistry/Microbiology	8 (15.4%)
Exercise/Kinesiology/Health	7 (13.5%)
Dietetics/Public Health	3 (5.8%)
Family Studies	9 (17.3%)
Food Science/Crop production	2 (3.8%)
Music	1 (1.9%)
Spanish	7 (13.5%)
**Residence**	
Northeast	3 (1.6%)
Mid-Atlantic	20 (10.7%)
South	19 (10.2%)
East North Central	49 (26.2%)
East South Central	22 (11.8%)
West North Central	2 (1.1%)
West South Central	12 (6.4%)
Mountain	28 (14.9%)
Pacific	29 (15.5%)
Prefer not to respond	3 (1.6%)

**Table 3:** Dietetic-Related Cultural Activities (*n* = 187).

Cultural Activities	No. of Responses (%)
Discussions	119 (63.6%)
Cultural-Competency Training	38 (20.3%)
Cultural Food Demonstration	70 (37.4%)
Nutrition Education Programs	44 (23.5%)
Interactive Case Studies	45 (24.1%)
Internship	6 (3.2%)
Non-interactive Case Studies	55 (29.4%)
Cultural Presentations	80 (42.8%)
Service Learning Activities	21 (11.2%)
Study Abroad	12 (6.4%)
Participated in 2–3 Cultural Activities	58 (31%)
Participated in 4–5 Cultural Activities	38 (20.3%)
Participated in six or more Cultural Activities	18 (9.6%)

**Table 4:** Factor loadings of general knowledge, attitudes, and beliefs (KAB) instrument.

Statement Items	Factor Loading	
	Knowledge	Attitudes & Beliefs
In my non-DPD (Didactic Programs in Dietetics) courses, I have been exposed to different cultures through assignments/activities/discussions.	0.51	
In my DPD courses, I have been exposed to more than one culture.	0.82	
In my DPD courses, the assignments/activities/discussions that I completed have exposed me to more than one culture.	0.74	
My knowledge about different cultures has increased.	0.80	
My knowledge about health issues among different cultures has increased.	0.70	
My ability to communicate about nutrition to different cultures has increased.	0.80	
My knowledge about food culture has increased.	0.76	
My understanding of cultural issues has increased.	0.80	
My understanding of the differences between ethnicity and culture has increased.	0.71	
My dietetic professors adequately address cultural issues.	0.70	
My dietetic professors are comfortable discussing cultural issues in the classroom.	0.58	0.36
My dietetic professors respect students from different cultures.	0.43	0.47
If I need more information about a patient’s culture, I would use resources available (e.g., books, videos, web-based resources).	0.89	
Overall, this DPD program has increased my knowledge and understanding to work with individuals from different cultures.	0.32	
My beliefs and attitudes are influenced by my culture.		0.80
My behaviors are influenced by my culture.		0.86
I often reflect on how culture affects beliefs, attitudes, and behaviors.		0.33
Before speaking with someone, I have pre-conceived notions about their culture.		0.42
I am less patient with people from different cultural backgrounds than my own.	0.41	0.44
I feel comfortable working with people from different cultural backgrounds than my own.	0.54	0.33
My cultural background influences how I behave in the classroom (asking questions, participating in groups, offering comments).		0.60
My own cultural beliefs may influence the decisions I make in patient simulations.		0.62
My dietetic professors have engaged in behaviors that noticeably made students from different cultural backgrounds feel excluded.		0.41
I respect the decisions of people from different cultural backgrounds than my own, even if I disagree.		0.47
If I need more information about a patient’s culture, I would feel comfortable asking the patient or family member.		0.49
I want my DPD program to teach more about different cultures.		0.33

**Table 5:** Average item scores and Cronbach alpha reliabilities for the general KAB instrument and dimensions (*n* = 187).

Dimensions	No. of Items	Mean (SD)	Cronbach Alpha
Knowledge	12	4.53 (0.89)	0.93
Attitudes & Beliefs	10	4.46 (0.41)	0.74
Total instrument	22	4.50 (0.65)	0.86

Note. All items were ranked on a 6-point Likert scale from “strongly disagree” to “strongly agree”.

**Table 6:** Multiple regression analyses of general KAB and predictors (*n* = 187).

Model	Predicting Variables	β	T	*p*-Value	
1 (Knowledge)	Constant		11.13	0.00	Multiple *R* = 0.30 $R_{Adj.}^{2}$ = 0.05 *MSE* = 0.87 *F-test* (7, 178) = 2.42 *p* = 0.02
	Gender	−0.01	−0.16	0.87	
	Age	−0.06	−0.62	0.54	
	Year in School	0.10	1.16	0.25	
	Race/Ethnicity	−0.01	−0.12	0.90	
	Study/Lived Abroad	0.09	1.18	0.24	
	Residence	−0.09	−1.17	0.24	
	No. of activities	0.26	3.54	0.001	
2 (Attitudes & Beliefs)	Constant		27.21	0.00	Multiple *R* = 0.29 $R_{Adj.}^{2}$ = 0.05 *MSE* = 0.40 *F-test* (7, 178) = 2.31 *p* = 0.03
	Gender	−0.09	−1.18	0.24	
	Age	0.01	0.16	0.87	
	Year in School	0.17	1.91	0.06	
	Race/Ethnicity	−0.04	−0.57	0.57	
	Study/Lived Abroad	−0.19	−2.49	0.01	
	Residence	−0.01	−0.07	0.94	
	No. of activities	0.07	0.91	0.37	

**Table 7:** Spearman correlations among general KAB scores and specific activities (*n* = 187).

Specific Cultural Activities within Dietetic Curriculum	Knowledge	Attitudes & Beliefs
	*R*	
Discussions	0.18 *	0.01
Cultural-competence training	0.26 *	−0.04
Food demonstrations	0.21 *	0.05
Nutrition education programs	0.23 *	−0.1
Interactive case studies	0.23 *	0.04
Internship	0.07	0.28
Non-interactive case studies	0.15 *	−0.06
Presentations	0.19 *	−0.01
Service-learning project	0.21 *	0.04
Study abroad	0.09	0.02

Note. * = *p* < 0.05.

## Supplementary Materials

The following are available online at https://www.mdpi.com/2072-6643/11/6/1202/s1, Table S1: Culture Survey.
