# Supplementary material for: Determining the Associations between Dietetic-Related Activities and Undergraduate Dietetic Students’ General Cultural Knowledge, Attitudes, and Beliefs
Source: Nutrients. 2019 May 28;11(6):1202. doi: 10.3390/nu11061202 (PMC6627802; doi:10.3390/nu11061202)
Supplement: Supplementary file 1 [file nutrients-11-01202-s001.pdf]

Culture-Survey:

For this survey, **culture** is defined as the learned, shared, and transmitted values, beliefs, norms and lifeways of a culture that guides thinking, decisions, and actions (Leininger, 1991). When responding to this survey consider that culture encompasses your ethnic or racial background, geographic location, religion, values, morals, and language (Office of Minority Health, 2005).

For the following 14 statements about culture, consider your DPD required professional courses (e.g. Community Nutrition, Medical Nutrition Therapy, Food Science, etc). Please indicate on a scale of 1 (strongly disagree) to 6 (strongly agree) your opinons.

	Strongly disagree (1)	Disagree (2)	Mildly Disagree (3)	Mildly Agree (4)	Agree (5)	Strongly Agree (6)
In my non-DPD courses, I have been exposed to different cultures through assignments/activities/discussions.						
Throughout my DPD courses, I have been exposed to more than 1 culture.						
In my DPD courses, the assignments/activities/discussions that I completed has exposed me to more than 1 culture.						
My knowledge about different cultures has increased.						
My knowledge about health issues among different cultures has increased.						
My ability to communicate about nutrition to different cultures has increased.						
My knowledge about food culture has increased.						
My understanding of cultural issues has increased.						
My understanding of the differences between ethnicity and culture has increased.						
My dietetic professors have engaged in behaviors that noticeably made students from different cultural backgrounds feel **excluded**.						
My dietetic professors are comfortable discussing cultural issues in the classroom.						
My dietetic professors adequately address cultural issues.						
My dietetic professors respect students from different cultures.						
Overall, this DPD program has increased my knowledge and understanding to work with individuals from different cultures.						

For this survey, **culture** is defined as the learned, shared, and transmitted values, beliefs, norms and lifeways of a culture that guides thinking, decisions, and actions (Leininger, 1991). When responding to this survey consider that culture encompasses your ethnic or racial background, geographic location, religion, values, morals, and language (Office of Minority Health, 2005).

Consider your beliefs, attitudes, and behaviors for the following 12 statements about culture. Please indicate on a scale of 1 (strongly disagree) to 6 (strongly agree) your opinions.

	Strongly disagree (1)	Disagree (2)	Mildly Disagree (3)	Mildly Agree (4)	Agree (5)	Strongly Agree (6)
My beliefs and attitudes are influenced by my culture.						
My behaviors are influenced by my culture.						
I often reflect on how culture affects beliefs, attitudes, and behaviors.						
Before speaking with someone, I have pre-conceived notions about their culture.						
My cultural background influences how I behave in the classroom (asking questions, participating in groups, offering comments).						
I respect the decisions of people from different cultural backgrounds than my own, even if I disagree.						
I am less patient with people from different cultural backgrounds than my own.						
I feel comfortable working with people from different cultural backgrounds than my own.						
My own cultural beliefs may influence the decisions I make in patient simulations.						
If I need more information about a patient's culture, I would use resources available (e.g. books, videos, web-based resources).						
If I need more information about a patient's culture, I would feel comfortable asking the patient or family member.						
I want my DPD program to teach more about different cultures.						

For the following 3 questions about culture, consider your DPD professional required courses (e.g. Community Nutrition, Medical Nutrition Therapy, Food Science).

1. How many spent more than 1 class period addressing culture?

    a. 0
    b. 1
    c. 2
    d. 3
    e. 4+
    f. Do not know

2. How many had an objective that focused on culture?

    a. 0
    b. 1
    c. 2
    d. 3
    e. 4+
    f. Do not know

3. Identify the type of activities/assignments you completed that focused-on culture (select all that apply):

    a. Classroom discussions
    b. Cultural-competency training
    c. Cultural food demo
    d. Designing a nutrition education program/lesson
    e. Interactive case studies (e.g. talking with a patient, role playing)
    f. Internship
    g. Non-interactive case studies (e.g. computer or paper)
    h. Presentations about a culture
    i. Service learning project in a community different from your own culture
    j. Study abroad immersive experience (working with the community)
    k. Other:

Demographics:

1. Please indicate your gender:
    a. Male
    b. Female
    c. Other
    d. Prefer not to respond

2. Please indicate your ethnicity
    a. American Indian or Alaska Native
    b. Asian
    c. Black, non-Hispanic
    d. Hispanic or Latino
    e. White, non-Hispanic
    f. Two or more ethnicities
    g. Other:
    h. Prefer not to respond

3. Please indicate the language(s) you speak fluently (select all that apply):
    a. Arabic
    b. Cantonese
    c. Mandarin
    d. English
    e. French
    f. German
    g. Korean
    h. Russian
    i. Spanish
    j. Tagalog
    k. Vietnamese
    l. Other:
    m. Prefer not to respond

4. Please indicate the language(s) you write/read fluently (select all that apply):
    a. Arabic
    b. Cantonese
    c. Mandarin
    d. English
    e. French
    f. German
    g. Korean
    h. Russian
    i. Spanish
    j. Tagalog
    k. Vietnamese
    l. Other:
    m. Prefer not to respond

5. Please indicate if you have study abroad and/or lived abroad
    a. Study abroad
    b. Lived abroad
    c. Both
    d. Neither
    e. Prefer not to respond

6. Please indicate your year in school:
    a. Freshman
    b. Sophomore
    c. Junior
    d. Senior
    e. Post-baccalaureate
    f. Prefer not to respond

7. Please indicate your age:
    a. 18-19 years
    b. 20-21 years
    c. 22-24 years
    d. 25 years and above
    e. Prefer not to respond

8. Please indicate if you are completing a double-major:
    a. Yes
    b. No

9. If yes, indicate your other major besides dietetics:

10. Please indicate if you are completing a minor(s):
    a. Yes
    b. No

11. If yes, indicate your minor(s):

12. Please indicate the area where you completed at least 75% of your DPD required courses:
    a. New England (Connecticut, Maine, Massachusetts, Rhode Island, Vermont)
    b. Mid-Atlantic (New Jersey, New York, Pennsylvania)
    c. South Atlantic (Delaware, Florida, Georgia, Maryland, North Carolina, South Carolina, Virginia, Washington DC, West Virginia)
    d. East North Central (Illinois, Indiana, Michigan, Ohio, Wisconsin)
    e. East South Central (Alabama, Kentucky, Mississippi, Tennessee)
    f. West North Central (Iowa, Kansas, Minnesota, Missouri, Nebraska, North Dakota, South Dakota)
    g. West South Central (Arkansas, Louisiana, Texas)
    h. Mountain (Arizona, Colorado, Idaho, Montana, Nevada, New Mexico, Utah, Wyoming)
    i. Pacific (Alaska, California, Hawaii, Oregon, Washington)
